# Recombinase-mediated cassette exchange (RMCE) system for functional genomics studies in *Mycoplasma mycoides*

**DOI:** 10.1186/s12575-015-0016-8

**Published:** 2015-03-02

**Authors:** Vladimir N Noskov, Li Ma, Stephen Chen, Ray-Yuan Chuang

**Affiliations:** Department of Synthetic Biology and Bioenergy, J. Craig Venter Institute, Rockville, MD USA

## Abstract

**Background:**

We have previously established technologies enabling us to engineer the *Mycoplasma mycoides* genome while cloned in the yeast *Saccharomyces cerevisiae,* followed by genome transplantation into *Mycoplasma capricolum* recipient cells to produce *M. mycoides* with an altered genome. To expand the toolbox for genomic modifications, we designed a strategy based on the Cre/loxP-based Recombinase-Mediated Cassette Exchange (RMCE) system for functional genomics analyses.

**Results:**

In this paper, we demonstrated replacement of an approximately 100 kb DNA segment of the *M. mycoides* genome with a synthetic DNA counterpart in two orientations. The function of the altered genomes was then validated by genome transplantation and phenotypic characterization of the transplanted cells.

**Conclusion:**

This method offers an easy and efficient way to manipulate the *M. mycoides* genome and will be a valuable tool for functional genomic studies, such as genome organization and minimization.

**Electronic supplementary material:**

The online version of this article (doi:10.1186/s12575-015-0016-8) contains supplementary material, which is available to authorized users.

## Introduction

High efficiency homologous recombination has played a critical role in yeast genetic studies and also has been widely used for other applications such as transformation-associated recombination for cloning of large pieces of DNA [1]. To extend this utility, we have developed a technology to build the genome of the bacterium, *M. mycoides* in yeast for the creation of the first synthetic cell [2]. Once cloned in yeast, the bacterial genome can be engineered by yeast genetic tools and subsequently transplanted into the recipient cell to produce a strain of *M. mycoides* with a modified genome [3]. Thus, this technique now provides a means for genome manipulation in *M. mycoides,* which is a genetically intractable bacterium. Furthermore, it also offers a great opportunity for research requiring whole genome constructions and engineering.

The Cre/loxP site-specific recombination method has been successfully used in a variety of genomic manipulations in both prokaryotic and eukaryotic organisms [4-7]. This system consists of two identical 34-bp loxP sites, where the recombination event takes place, and a Cre recombinase, which catalyzes the recombination between the two loxP sites [8]. The Cre/loxP system has been used to perform a variety of genomic modifications including insertions, deletions, translocations and inversions at specific sites in the genome. In order to enhance the repertoire of tools available for genome engineering, we developed a method using the RMCE system [9] in the yeast *S. cerevisiae*. RMCE allows unidirectional integration of a DNA fragment from one molecule into a pre-determined genomic locus. It involves double recombination events, catalyzed by a recombinase, between two hetero-specific loxP sites within a genomic target site and a plasmid donor DNA. To demonstrate its utility, we swapped a 100-kb segment in the *M. mycoides* genome containing 84 annotated genes with its counterpart synthetic DNA segment, and also placed it in an inverted orientation. We found that the genome containing the 100-kb inverted segment was able to boot up in the recipient cells to produce a new *M. mycoides* strain with a similar growth phenotype to that of the wild type genome.

## Materials and methods

### Yeast strains, media, and transformation

The *S. cerevisiae* yeast strains used here were VL6-48 (*MATα, his3Δ200, trp1Δ1, ura3-52, lys2, ade2–101, met14*), W303-1a (*MATa leu2-3,112 trp1-1 can1-100 ura3-1 ade2-1 his3-11,15*) and both VL6-48 and W303-1a containing the 1.08–mega–base pair *M. mycoides* genome [10]. Yeast cells were grown in standard rich medium containing glucose (YEPD) or galactose (YEPG); or in synthetic minimal medium containing dextrose (SD) [11]. Yeast transformation was carried out by either Lithium-acetate [12] or spheroplast [13] procedure.

### Vectors

**Figure 1:**
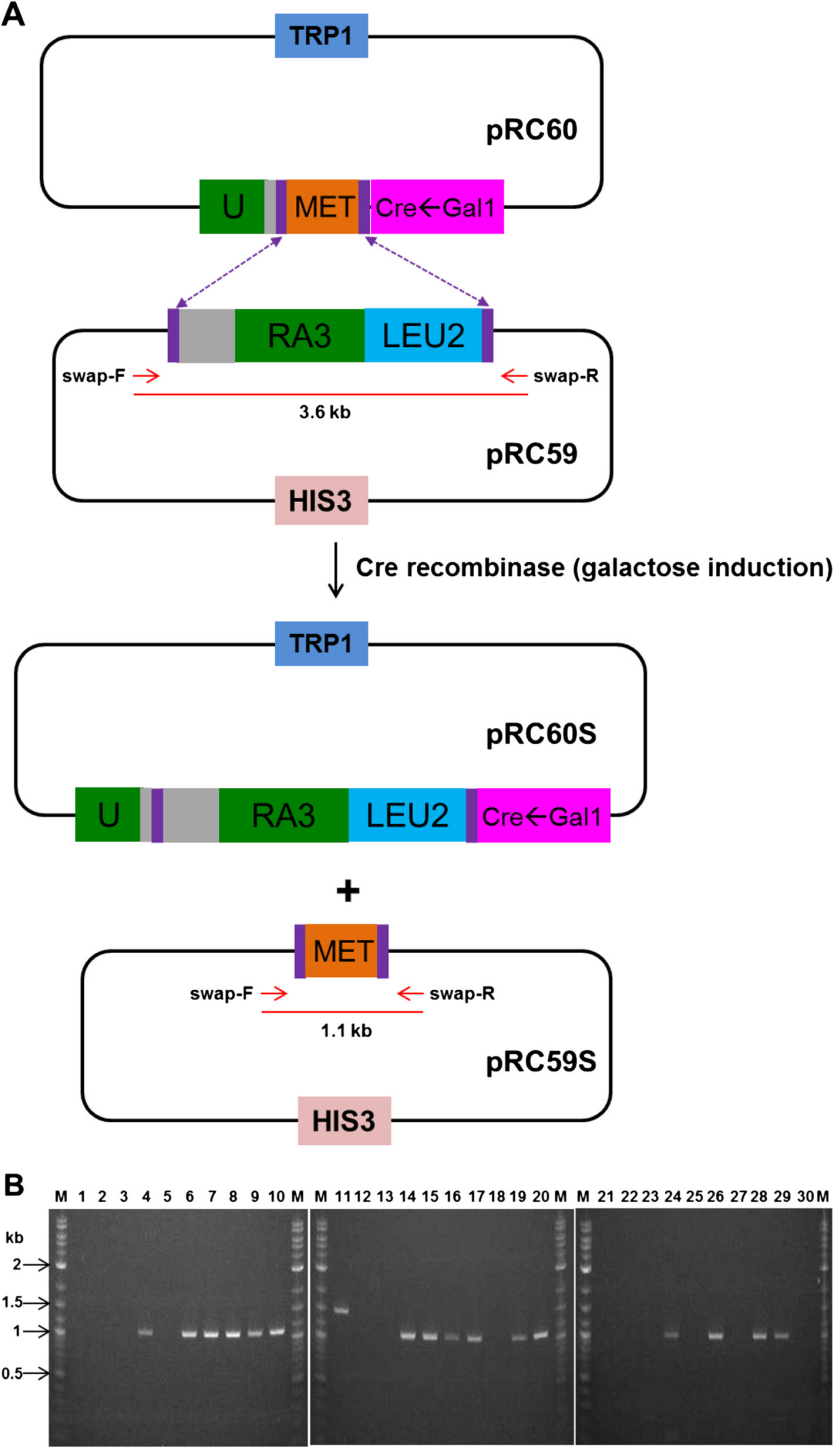
**Design of the Recombinase-Mediated Cassette Exchange. (A)** The scheme of RMCE between the recipient plasmid (pRC59) and the donor plasmid (pRC60). pRC59 contains a floxed cassette, consisting of the truncated 3′URA3 gene and the yeast LEU2 marker; and pRC60 contains the 5′URA3 gene, a floxed yeast MET14 ORF, and the Cre recombinase gene under the GAL1 inducible promoter. The gray color indicates the actin intron. The purple bars represent 34 bp hetero-specific loxP mutants where cassette exchange takes place, marked by broken arrows. The cassette exchange was performed by growing the yeast harboring two plasmids in medium containing galactose for 24 hours, followed by the selection of uracil prototrophs on SD-Uracil plates. The cassette exchange would produce two plasmids, pRC59S and pRC60S. The exchange event was evaluated by PCR using primers (swap-F and swap-R) indicated by red arrows. pRC59S allows the amplification of a 1.1 kb product, in contrast to the 3.6 kb product amplified from the parental pRC59. **(B)** PCR screening for cassette exchange. Cassette exchange was performed in two yeast strains, W303a and VL6-48. Fifteen colonies from each strain were analyzed by PCR. Lanes 1 to 15: W303a strain; and lanes 16 to 30: VL6-48 strain; M: DNA marker.

The RMCE system described in this paper consists of two plasmids, pRC59 and pRC60. The plasmid pRC59 contains a cassette, called a landing pad, which marks the target site of the genome and the pRC60 vector is a donor plasmid, which carries DNA for cassette exchange (Figure 1A). Construction of these two plasmids is described in the Additional file 1: Supplementary data.

### Transformation-Associated Recombination (TAR) cloning of the 100 kb DNA segment

A 7.5 kilobase (kb) TAR cloning vector was generated by PCR amplification of pRC60 using two primers (5′- *ACTAATAATAAAACATTTATATACTTAATGAATAAATATAATTAG*TACCGTTCGTATAATGTATGC-3′ and 5′-*ATTTTAAAATTTATGTAATTTATTAATTTTTATCTTTATAATATA*TACCGTTCGTATATGGTTTCT-3′) and the Phusion Hot Start High-Fidelity DNA polymerase with HF buffer (New England Biolabs; NEB) according to the manufacturer’s instructions with modifications. The reactions were supplemented with 1 mM additional MgCl_2_, and the products were annealed at 64°C and extended for 1 min per kb. Two 50-bp TAR cloning hooks (italicized) are homologous sequences necessary for recombination [13]. Approximately 40 ng of vector was co-transformed with 1 μg of sheared *M. mycoides*-syn1.0 genomic DNA [2] into VL6-48 spheroplasts. Transformants were selected on SD minus tryptophan.

### Insertions of the landing pad

The pRC59 vector was used as the DNA template for production of two landing pad cassettes (forward and reverse). A 3.4 kb of the forward landing pad cassette was PCR-amplified using Phusion Hot Start High-Fidelity DNA polymerase (New England Biolabs; NEB) according to the manufacturer’s instructions. Primers (5′-AAATCAAGATCTTTTGGCAGCATATTTCTACTCTTTTTCTATTTATTAGT TACCGTTCGTATAAGAAACCA-3′ and 5′-TGATTACAACTAGTTTAACAATTTATTAAAAAACTTCGTAAAAACGAAGT TACCGTTCGTATAGCATACAT-3′) were used for production of the forward cassette and primers (5′-TAAGTTCTTATGATTACAACTAGTTTAACAATTTATTAAAAAACTTCGTAAAAACGAAGT TACCGTTCGTATAAGAAACCA-3′ and 5′-TTGTAGCTTAAAATCAAGATCTTTTGGCAGCATATTTCTACTCTTTTTCTATTTATTAGTTACCGTTCGTATAGCATACAT-3′) were used for production of the reverse cassette. Approximately 1 μg of PCR product was transformed into the yeast strain W303 containing the *M. mycoides* genome. Transformants were selected on SD minus leucine. All primers were purchased from Integrated DNA Technologies (Coralville, IA, USA).

### Colony PCR

Yeast colonies were patched to an appropriate selection medium and grown overnight at 30°C. Approximately 1 μl of cell mass was then picked up by pipette tip and twirled in a 0.5 ml PCR tube containing 10 μl of the zymolyase solution [10 μl of sterile water + 0.5 μl of 10 mg/ml of zymolyase 20T (ICN Biochemicals)]. The tube was incubated at 37°C for 1 hour, followed by 15 min incubation at 98°C. 1 μl of zymolyase-treated cells was analyzed by PCR using the QIAGEN Fast Cycling PCR Kit, according to the manufacturer's instructions.

### Restriction analysis of *M. mycoides* genome

The detailed preparation of genomic DNA in agarose plugs from yeast and *M. mycoides* cells was described previously [2,10]. Once prepared, yeast plugs were loaded onto a 1% Tris-acetate-EDTA agarose gel and electrophoresis was performed at 4.5 V per cm for 2 hours to remove yeast genomic DNA from the plugs (the circular *M. mycoides* genomes remained in the plug) [14]. To analyze the genomic structure by restriction digestion, half of an agarose plug was washed once with 1 ml of 0.1X Wash Buffer (Bio-Rad CHEF Genomic DNA Plug Kit) for 1 hour followed by 1 hour of washing with the same buffer plus 0.5 mM phenylmethylsulfonyl fluoride (Sigma, St. Louis, MO). Plugs were then equilibrated with 1 ml of 1X Buffer 3 (NEB) for 1 hour. The genomic DNA was digested with 50 units of the restriction enzymes BssHII in 250 μl of 1X buffer 3 for 5 hours at 37°C. Following incubation, plugs were subjected to pulsed-field gel electrophoresis (CHEF DRIII, Bio-Rad). Pulse times were ramped from 20 to 50 seconds for 16 hours at 6.0 V/cm. All restriction enzymes were purchased from NEB.

## Results

### Design of the Recombinase-Mediated Cassette Exchange (RMCE)

To perform unidirectional cassette exchange, four hetero-specific loxP sites were adapted in our RMCE system to prevent potentially promiscuous recombination [15].

The cassette exchange event is commonly screened by functional restoration of a reporter gene. We engineered a novel reporter gene, the yeast URA3 gene with a modified yeast ACTIN intron where a 34 base-pair mutant loxP site was inserted between the 5′ splice site and the branch point of the intron (Additional file 1: Supplementary sequence). The modified URA3 gene was then split into the recipient plasmid, pRC59 and the donor plasmid, pRC60 (Figure 1A and Additional file 1: Figure S1). Both pRC59 and pRC60 contain essential elements for our RMCE system. In the pRC59 plasmid, a 3.4 kb cassette (dubbed a landing pad) contained (from 5′ to 3′ end) the loxm2/66, the 3′ truncated URA3, the LEU2 marker, and the lox71, and was used to mark the target site. The pRC60 included the yeast TRP1 marker, the Cre recombinase gene under the GAL1 inducible promoter, the 5′ modified URA3 gene, and two hetero-specific loxP sites (loxm2/71 and lox66) encompassing DNA sequence for exchange (here the yeast MET14 ORF is present). To test recombinase-mediated cassette exchange, both pRC59 and pRC60 plasmids were transformed into two yeast strains (W303a and VL6-48 respectively), and selected for histidine and tryptophan prototrophs. Growing the histidine and tryptophan positive clones in the galactose medium induced the expression of Cre recombinase. If double recombination took place, we anticipated that the MET14 (609 bp) from the pRC60 would be swapped with the landing pad, resulting in production of the pRC59S and the pRC60S (Figure 1A). After 24 hours of galactose induction, cells were streaked out on SD-Uracil plates. Fifteen colonies from each transformation were PCR-analyzed using primers (swap-F and swap-R) for one of the cassette exchange products, pRC59S (Figure 1A). The primers would generate a 1.1 kb PCR product from the pRC59S and a 3.6 kb product from the pRC59. We found that the frequency of correct exchange in both yeast strains is greater than 50% (8/15). On the other hand, six uracil positive clones did not yield any PCR product and one produced a PCR product with incorrect size (Figure 1B). We reasoned that it might be due to an incomplete cassette exchange, which means only a single recombination occurred on the URA3 site leading to a joining of two circular plasmids into a larger hybrid. Given the high efficiency of cassette exchange obtained above, we applied this system to conduct a genome-scale manipulation on the *M. mycoides* genome in yeast.

### Construction of a semi-synthetic genome

**Figure 2:**
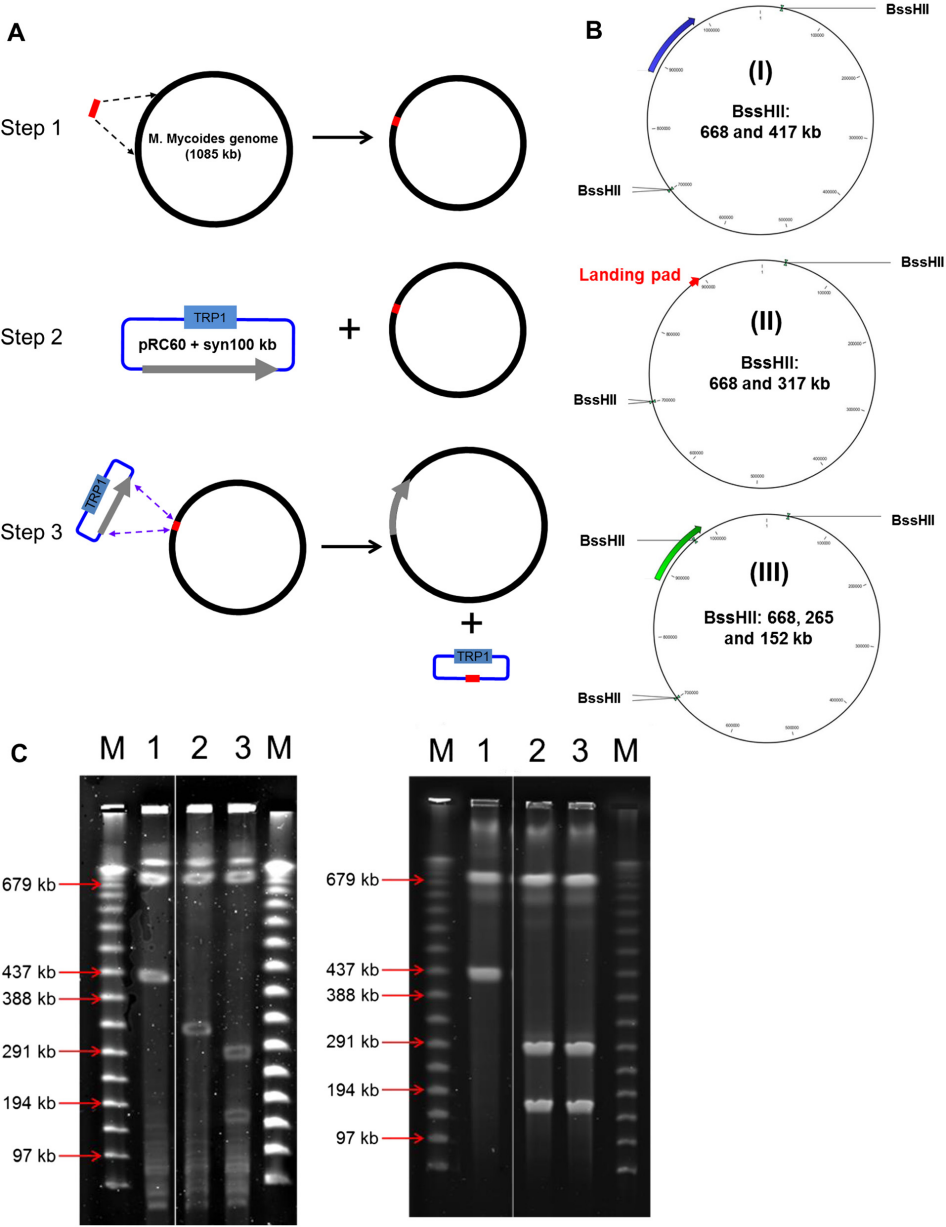
**Construction of a semi-synthetic** ***M. mycoides*** **genome by RMCE. (A)** Schematic diagram of three steps of RMCE. First step: replacement of a 100 kb segment with the landing pad (red bar) via homologous recombination (broken arrows) in yeast. Second step: transformation of the donor plasmid pRC60 carrying the 100 kb synthetic segment (gray solid arrow) to the yeast containing the landing pad genome. Third step: introduction of the synthetic DNA segment into the genome via the loxP sites catalyzed by Cre recombinase, represented by purple arrows. **(B)** BssHII restriction enzyme maps of *M. mycoides* genomes:(I) Wild type, (II) the landing pad replacement, and (III) the semi-synthetic genomes. Three BssHII sites exist in wild type *M. mycoides* genome and two sites are overlapped with each other. The target 100 kb segment (blue arrow) in the genome (I) was replaced with the landing pad (red arrow) shown in genome (II) which was subsequently exchanged with the synthetic counterpart (green arrow) shown in genome (III). An additional BssHII site exists in the synthetic DNA segment, closest to the 3′end of the segment. **(C)** Contour-clamped homogeneous electric field (CHEF) electrophoresis analysis of BssHII-digested *M. mycoides* genomes. *M. mycoides* genome purified from yeast was digested with BssHII shown in the left panel, lane 1: wild type, lane 2: the landing pad replacement, and lane 3: the semi-synthetic genomes. *M. mycoides* genomes purified from bacterial transplants were digested with BssHII shown in the right panel, lane 1: wild type, lane 2: semi-synthetic clone 1 and lane 3: semi-synthetic clone 2. M: 50 kb lambda DNA ladder.

To demonstrate the feasibility of this recombinase-based system for genomic manipulation, we swapped an approximately 100 kb segment (from MMCAP2_0749 to MMCAP2_0832) of the *M. mycoides* genome with its synthetic DNA counterpart to produce a semi-synthetic genome. Since two loxP sites would remain at both ends of the target region after cassette exchange, they were selected to be located outside of the ORF. The procedure included three steps illustrated in Figure 2A. First, the target DNA segment in the *M. mycoides* genome was replaced with the landing pad via homologous recombination in yeast. Second, the synthetic DNA segment carried by the donor plasmid pRC60 was transformed into the yeast strain harboring the genome with an insertion of the landing pad (in step 1). Third, the synthetic DNA segment was exchanged with the landing pad via the loxP sites catalyzed by Cre recombinase.

A replacement of the landing pad cassette with the target region was screened by PCR using primers flanking both ends of the cassette (data not shown) and was later further characterized by analysis of restriction enzyme digestion (see below). The 100 kb synthetic DNA was cloned in the donor plasmid pRC60 by the transformation-associated recombination (TAR) method (see Materials and methods) and analyzed by gel electrophoresis to estimate the length of the insert (Additional file 1: Figure S2). After transformation of the donor plasmid carrying the synthetic DNA segment into the strain containing the landing pad inserted genome, the cassette exchange was induced by galactose for 24 hours, followed by selection for uracil protorophy. The cassette exchange was evaluated by PCR at both junctions of the target region. We found that the frequency of correct exchange is greater than 50%, which is similar to that of the testing plasmid described above (data not shown). Compared with the native *M. mycoides* genome, we further performed restriction analysis of the genomic structure of the landing pad insertion and the semi-synthetic product (Figure 2B). The native *M. mycoides* genome digested with restriction enzyme, BssHII, yielded 668 kb and 417 kb products, whereas the landing pad inserted genome digested with the same enzyme produced 668 kb and 317 kb products (Figure 2C, lane 1 and 2 in left panel). On the other hand, since the synthetic segment contains a BssHII recognition site (Figure 2B), the semi-synthetic genome digested with the same enzyme would produce 668 kb, 224 kb, and 193 kb products (Figure 2C, lane 3 in the left panel). Next, we carried out transplantation of all three genomes (the native, the landing pad inserted, and the semi-synthetic genome). As expected, we found only the native and semi-synthetic genome gave rise to transplant colonies. The genomic structures from the two transplanted colonies were analyzed by BssHII as described above. The restriction pattern was consistent with that of genome constructed in yeast (Figure 2C, lane 2 and 3 in the right panel).

### Production of a re-structured *M. mycoides* genome

**Figure 3:**
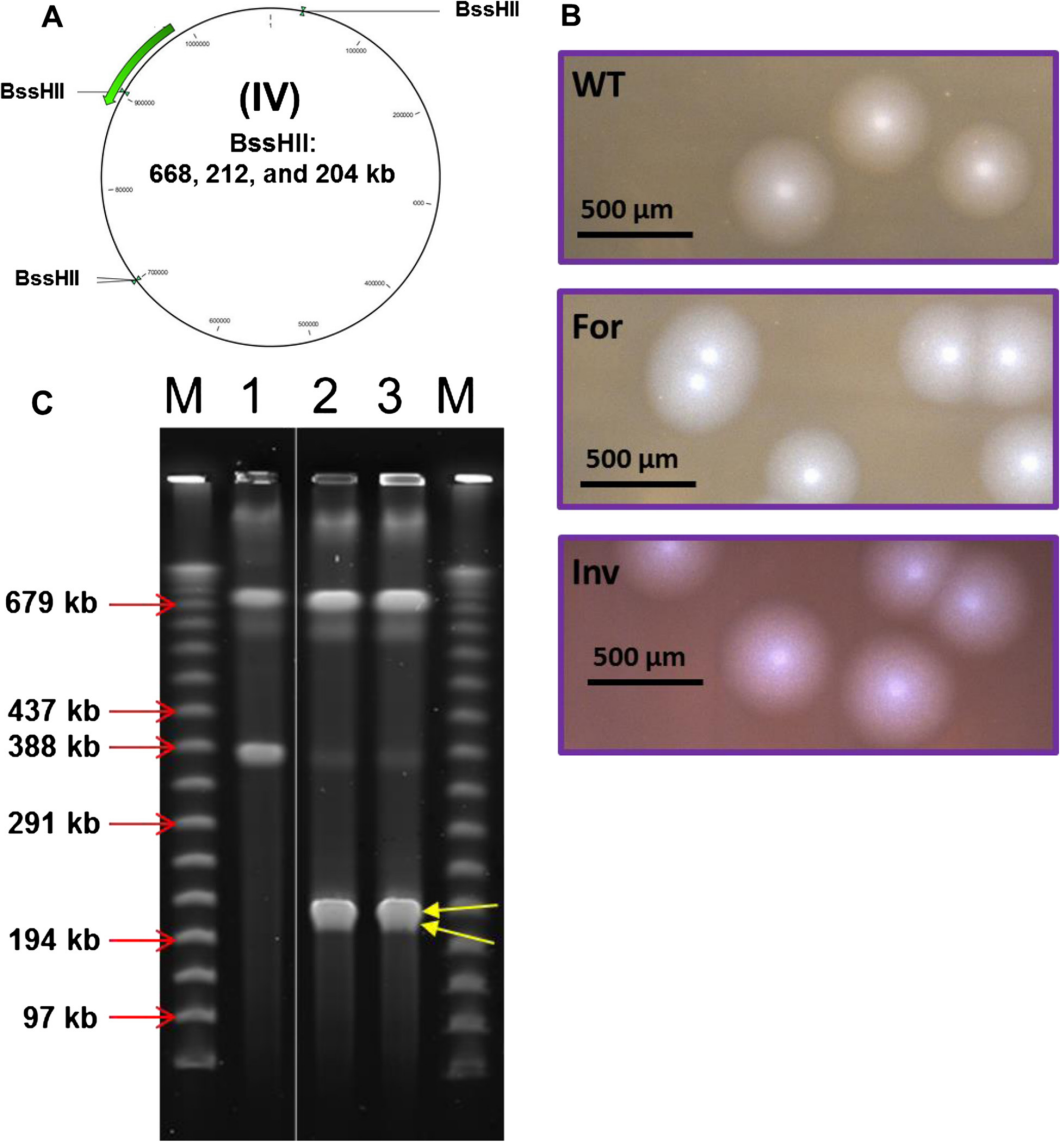
**Construction of a** ***M. mycoides*** **with re-structured genome. (A)** BssHII restriction enzyme map of the *M. mycoides* genome with an inversion of the 100 kb synthetic DNA. **(B)** Transplantation colonies from wild type and two semi-synthetic genomes. Transplant colonies by genomes from yeast clones (from left to right): wild type genome (WT), the 100 kb-forward insertion genome (For) and the 100 kb-inverted insertion genome (Inv). **(C)** CHEF electrophoresis analysis of BssHII-digested *M. mycoides* genomes. *M. mycoides* genomes purified from bacterial transplants were digested with BssHII, lane 1: WT genome, lane 2: Inv genome (clone 1), and lane 3: Inv genome (clone 2). Two DNA fragments (212 and 204 kb) co-migrated shown in lane 2 and 3, indicated by yellow arrows. M: 50 kb lambda DNA ladder.

Finally, we demonstrated the application of the RMCE system in a study of the organization of the *M. mycoides* genome. A total of 84 annotated genes within the 100 kb synthetic DNA described above were re-positioned in the genome. To this aim, we reinserted the landing pad to the same target segment in the opposite direction. Since the direction of the cassette exchange is determined by the hetero- specific loxP sites, the synthetic piece cloned in the same donor plasmid would be swapped to the same locus in an inverted direction (Figure 3A). The yeast clone containing the *M. mycoides* genome with the inverted insertion of the landing pad was isolated by PCR screening (data not shown) and then transformed with the same donor plasmid carrying the synthetic DNA. After induction of the cassette exchange, we conducted PCR screening to isolate yeast clones that contain a correct exchange product. Approximately 50% of positive clones were obtained. Next, the functionality of the re-structured genome was examined by genome transplantation. We found that the semi-synthetic genome with the inversion of the 100 kb DNA segment was able to produce transplant colonies (Figure 3B). Compared to the size of colonies transplanted with native genomes, there were no obvious differences. Finally, we analyzed the genomic structure by restriction using the enzyme BssHII to confirm that the synthetic segment was inserted in inverse orientation. Since it was swapped back to the landing pad in the opposite direction, the genome digested by BssHII should produce 668 kb, 212 kb, and 204 kb products. We found, indeed, that the new BssHII–digested pattern was observed from the two transplanted clones, as expected (Figure 3C, lane 2 and 3).

## Discussion

We presented here a simple and highly effective RMCE–based method for genetic engineering in yeast. One important feature in this system is the reconstitution of the reporter marker composed of two truncated 5′ and 3′ yeast URA3 genes spilt by a modified yeast actin intron. In this case, the selection of the cassette exchange event is tightly correlated with uracil prototrophy. In contrast, our initial design of the reporter marker consisted of a promoter and promoter-less gene. We found that leaky expression of promoter-less reporters, including the yeast LEU2 and the geneticin resistant gene, KanMX4 resulted in a high background in the cassette exchange screening (data not shown).

We previously reported the synthesis of the 1,078 kb *M. mycoides*-syn1.0 genome, followed by genome transplantation to produce the first synthetic cell [2]. The construction of the synthetic genome was performed in three hierarchical stages by transformation and homologous recombination in yeast, starting with assembling 10 kb, to 100 kb, to the complete 1,078 kb genome. To verify the functionality of the synthetic 100 kb intermediates, semi-synthetic genomes were assembled from a synthetic piece and 10 native ones, followed by transplantation analysis [2]. While this approach enabled us to build a variety of genomes for testing, the efficiency of complete assembly was very low (~2 to 5%). Furthermore, the procedure of genome assembly was tedious and time-consuming since it involved isolation of large pieces of DNA from yeast and purification of DNA fragments by electrophoresis [14]. By using the swapping system described above, we showed that the semi-synthetic genome could be easily constructed (50% efficiency) and that the effort and time were significantly reduced. In addition, we found that cloning or assembly of an insert DNA to the donor vector could be conducted directly in a yeast strain harboring the *M. mycoides* genome with the insertion of the landing pad where the cassette exchange could be performed. This further simplifies the procedure of genome construction without the steps of the donor DNA preparation and transformation (unpublished result). We also demonstrated that this swapping system can facilitate the study of genome organization by inverting the same synthetic piece in the opposite orientation. Previous studies of genome rearrangements were conducted by creation of inversion through insertion of two loxP sites flanking the target region of interest where the Cre recombinase triggered the rearrangement [16], and a similar approach using the Frt/Flp recombinase was also reported [17]. However, these approaches only allow a one-time inversion event.

In conclusion, the RMCE swapping method is a robust genomic engineering tool that offers great potential for accurate genome manipulation. With the advantage of a genome cloned in yeast, any genome modification can be first created without any concerns of phenotypic consequences. This allows genomic engineering to be more flexible. For instance, after replacement of the landing pad with a DNA segment of interest, the swapping method allows repeatable, yet precise insertion of design DNA into the target locus followed by transplantation to characterize functionality of altered genomes. These designed pieces can be built with a specific rearrangement of genes or operons to address genomic organization questions or with the removal of candidates of non-essential genes to study genome minimization (in preparation).

## Additional file

Additional file 1:
**Supplementary data is available online.**
