# Supplementary material for: Recombinase-mediated cassette exchange (RMCE) system for functional genomics studies in Mycoplasma mycoides
Source: Biol Proced Online. 2015 Mar 2;17:6. doi: 10.1186/s12575-015-0016-8 (PMC4359775; doi:10.1186/s12575-015-0016-8)
Supplement: Additional file 1: — Supplementary data is available online. [file 12575_2015_16_MOESM1_ESM.pdf]

Recombinase-mediated cassette exchange (RMCE) system for functional genomics studies in *Mycoplasma mycoides*

Supplementary Materials

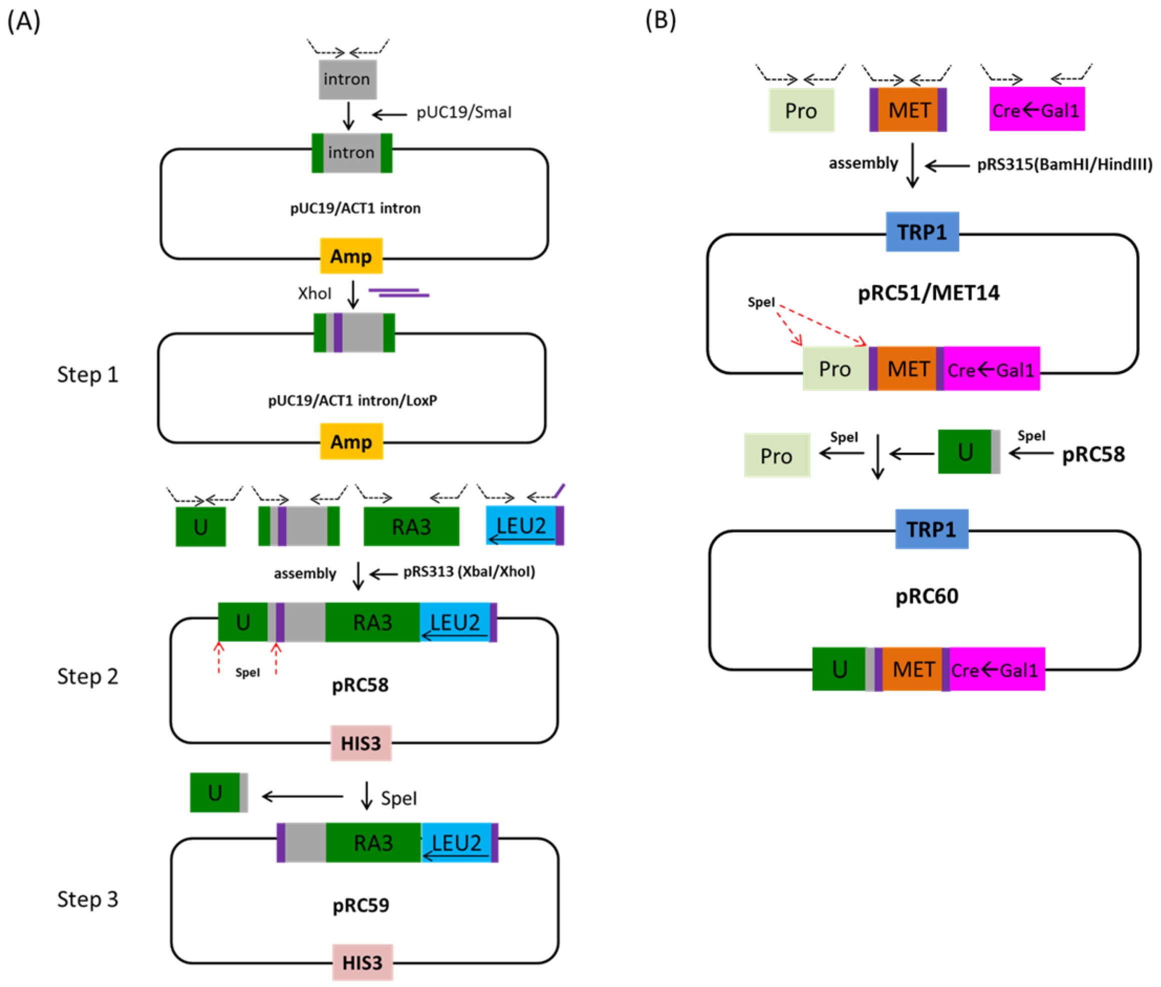

Supplementary Figure 1. Plasmid constructions. **(A)** Construction of the recipient plasmid pRC59 in three steps: (1) insertion of a 34-base pair (bp) mutant LoxP site, m2/Lox66 to the yeast actin intron. A 309 base-pair of the actin intron was PCR-amplified from genomic DNA of yeast strain W303a using forward primer 5'-ATCCCGGGGTTGCTGCCAAGCTATTTAATATCAGTATGTTCTAGCGCTTGCAC-3' and reverse primer 5'-ATCCCGGGCACAAGTTTGTTTGCTTTTCGTGCACTAAACATATAATATAGCAACAAAAAG-3'. The 25 nucleotides underlined on both primers, which were introduced to the intron PCR product, provide homology sequence for an assembly of a URA3 gene with the modified actin intron (described in the step 2). The PCR product was then digested with a restriction enzyme SmaI and cloned into SmaI site of the pUC19, resulting in production of the pUC19/ACT1 intron. Two oligonucleotides with 5' phosphorylation (5'-Pi-TCGAGACTAGTTACCGTTCGTATAAGAAACCATATACGAAGTTATC-3' and 5'-Pi-TCGAGATAACTTCGTATATGGTTTCTTATACGAACGGTAACTAGTC-3') were annealed to form a double-stranded linker containing the 34 bp mutant LoxP site (underlined) with XhoI cohesive ends that subsequently was inserted into the XhoI site located at 63 bp-

downstream of the actin intron in the pUC19/ACT1 intron to produce the pUC19/ACT1 intron/loxP. A correct orientation of the LoxP insertion was identified by sequencing (data not shown). (2) Construction of the pRC58 plasmid containing the yeast LEU2 gene and a URA3 gene engineered with the modified Actin intron. The LEU2 gene (2.25 kb) was PCR-amplified from the plasmid pRS315 (ref) using primer pair (5'-CAATTTAATTATATCAGTTATTACCCTATATCGACTACGTCGTAAGGCC-3' and 5'-CCTCACTAAAGGGAACAAAAGCTGGGTACCGGGCCCCCCCTCGAGTACCGTTCGTATAGCATACATTATACGAAGTTATGCATGCTCACACCGCATATCGACCCTC-3') and another 34 bp of mutant LoxP, LoxP71, was embedded to the reverse primer (underlined) so that it could be introduced to the upstream of the LEU2 gene after PCR amplification. A 320 bp of 5' portion and an 810 bp of 3' portion of the URA3 gene was PCR-amplified from the pRS316 (ref) using primer pairs (5'-ACTCACTATAGGGCGAATTGGAGCTCCACCGCGGTGGCGGCCGCTCTAGAACTAGTGATTGTACTGAGAGTGCACC-3' and 5'-ATCCCGGGCACAAGTTTGTTTGCTTTTCGTGCA CTAAACATATAATATAGCAACAAAAAG-3') and (5'-TTTTGTTGCTATATTATATGTTTAGTGCACGAAAAGCAAACAAACTTG-3' and 5'-CGGCCTTACGACGTAGTCGATATAGGGTAATAACTGATATAATTAAATTG-3'), respectively. To produce the plasmid pRC58, the four pieces of DNA including the 5' and 3' truncated URA3 gene, the modified Actin intron (released from the plasmid pUC19/ACT1 intron/loxP by SmaI) and the LEU2 gene, were co-transformed with a linearized pRS313 vector (ref) generated by restriction enzyme digestions of XbaI and XhoI, into yeast to assemble the pRC58 circular plasmid via unique 50 bp overlaps flanking each piece for homologous recombination and selected for uracil autotroph. (3) Construction of a truncated URA3 gene. Plasmid was recovered from uracil autotrophic yeast colonies in step 2 and then transformed to E. coli. Plasmid DNA was prepared using the Qiagen DNA MiniPrep Kit and correct constructs were confirmed by sequencing (data not shown). To produce the 5' truncated URA3 gene, 320 bp of the 5' URA3 portion was removed using restriction enzyme SpeI followed by self-ligation to produce the pRC59. **(B)** Construction of donor plasmid pRC60 in two steps. First, a pRC51-MET14 was made by assembling 4 piece DNA fragments through unique 50–bp overlaps flanking each piece in yeast in the same manner as described above. These DNA fragments are: (a) a 661-bp of 5' un-translated region (5'UTR) of the yeast LEU2 open reading frame (ORF) PCR-amplified from the pRS315 (ref) using primers (5'-GACTCACTATAGGGCGAATTGGAGCTCCACCGCGGTGGCGGCCGCTCTAGAACTAGTACTAGTATATCGACCCTCGAGGAGAAC-3' and 5'-ATCTGCAGTACCGTTCGTATATGGTTTCTTATACGAAGTTATACTAGTAGACATTAGAATGGTATATCCTTG-3'), (b) mutant loxP site-flanked the MET14 ORF (609-bp) PCR-amplified from yeast w303a genome using primers (TCTACTAGTATAACTTCGTATAAGAAACCATATACGAACGGTACTGCAGATGGCTACTAATATTACTTGGCA and GGCATGCATAACTTCGTATAGCATACATTATACGAACGGTACTGCAGTTACAAATGCTTACGGATGATTTTT), (c) a 1.5-kb of the Cre-recombinase under control of the GAL1 promoter PCR-amplified from the pBS185 (ref), and (d) the linearized pRS315 vector (ref) produced by double restriction digestion of BamHI and HindIII. Plasmid was recovered from tryptophan autotrophic yeast colonies and then transformed to E. coli. Second, the 661-bp of 5'UTR DNA fragment from the pRC51-MET14 was replaced with the SpeI fragment of the 320 bp of 5' URA3 described above, resulting in plasmidpRC60.

All PCR was performed using Phusion Hot Start High-Fidelity DNA (New England Biolabs; NEB) according to the manufacturer's instructions. All primers were purchased from Integrated DNA Technologies, IDT.

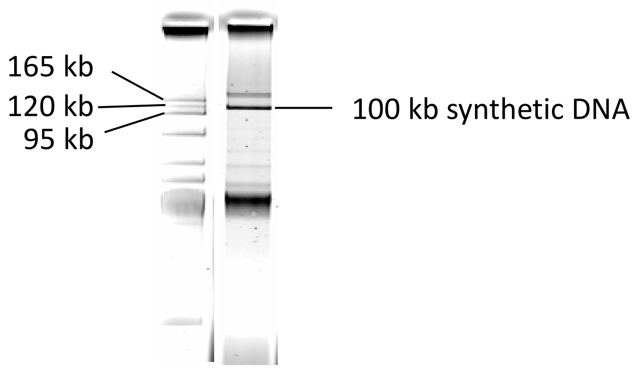

Supplementary Figure 2. Analysis of 100-kb synthetic DNA purified from yeast. The 100-kb synthetic DNA fragment was TAR-cloned from the synthetic genome (ref). The 107-kb circles (100-kb insert plus 7-kb pRC60) were separated from the linear yeast chromosomal DNA on a 1% agarose gel by applying 4.5 V/cm for 3 hours. M indicates the BAC-Tracker supercoiled DNA ladder (Epicentre).

Supplementary sequences

pRC58:

TCGCGCGTTTCGGTGATGACGGTGAAAACCTCTGACACATGCAGCTCCCGGAGACGGTCA
CAGCTTGTCTGTAAGCGGATGCCGGGAGCAGACAAGCCCGTCAGGGCGCGTCAGCGGGT
GTTGGCGGGTGTCGGGGCTGGCTTAACTATGCGGCATCAGAGCAGATTGTACTGAGAGTG
CACCATAATTCCGTTTTAAGAGCTTGGTGAGCGCTAGGAGTCACTGCCAGGTATCGTTTGA
ACACGGCATTAGTCAGGGAAGTCATAACACAGTCCTTTCCCGCAATTTTCTTTTTCTATTAC
TCTTGGCCTCCTCTAGTACACTCTATATTTTTTTATGCCTCGGTAATGATTTTCATTTTTTTTT
TTCCACCTAGCGGATGACTCTTTTTTTTTCTTAGCGATTGGCATTATCACATAATGAATTATA
CATTATATAAAGTAATGTGATTTCTTCGAAGAATATACTAAAAAATGAGCAGGCAAGATAAA
CGAAGGCAAAGATGACAGAGCAGAAAGCCCTAGTAAAGCGTATTACAAATGAAACCAAGAT
TCAGATTGCGATCTCTTTAAAGGGTGGTCCCCTAGCGATAGAGCACTCGATCTTCCCAGAA
AAAGAGGCAGAAGCAGTAGCAGAACAGGCCACACAATCGCAAGTGATTAACGTCCACACA
GGTATAGGGTTTCTGGACCATATGATACATGCTCTGGCCAAGCATTCCGGCTGGTCGCTAA
TCGTTGAGTGCATTGGTGACTTACACATAGACGACCATCACACCACTGAAGACTGCGGGAT
TGCTCTCGGTCAAGCTTTTAAAGAGGCCCTACTGGCGCGTGGAGTAAAAAGGTTTGGATCA
GGATTTGCGCCTTTGGATGAGGCACTTTCCAGAGCGGTGGTAGATCTTTCGAACAGGCCG
TACGCAGTTGTCGAACTTGGTTTGCAAAGGGAGAAAGTAGGAGATCTCTCTTGCGAGATGA
TCCCGCATTTTCTTGAAAGCTTTGCAGAGGCTAGCAGAATTACCCTCCACGTTGATTGTCT
GCGAGGCAAGAATGATCATCACCGTAGTGAGAGTGCGTTCAAGGCTCTTGCGGTTGCCAT

AAGAGAAGCCACCTCGCCCAATGGTACCAACGATGTTCCCTCCACCAAAGGTGTTCTTATG
TAGTGACACCGATTATTTAAAGCTGCAGCATACGATATATATACATGTGTATATATGTATACC
TATGAATGTCAGTAAGTATGTATACGAACAGTATGATACTGAAGATGACAAGGTAATGCATC
ATTCTATACGTGTCATTCTGAACGAGGCGCGCTTTCCTTTTTTCTTTTTGCTTTTTCTTTTTTT
TTCTCTTGAACTCGACGGATCATATGCGGTGTGAAATACCGCACAGATGCGTAAGGAGAAA
ATACCGCATCAGGAAATTGTAAACGTTAATATTTTGTTAAAATTCGCGTTAAATTTTTGTTAA
ATCAGCTCATTTTTTAACCAATAGGCCGAAATCGGCAAAATCCCTTATAAATCAAAAGAATA
GACCGAGATAGGGTTGAGTGTTGTTCCAGTTTGGAACAAGAGTCCACTATTAAAGAACGTG
GACTCCAACGTCAAAGGGCGAAAAACCGTCTATCAGGGCGATGGCCCACTACGTGAACCA
TCACCCTAATCAAGTTTTTTGGGGTCGAGGTGCCGTAAAGCACTAAATCGGAACCCTAAAG
GGAGCCCCCGATTTAGAGCTTGACGGGGAAAGCCGGCGAACGTGGCGAGAAAGGAAGGG
AAGAAAGCGAAAGGAGCGGGCGCTAGGGCGCTGGCAAGTGTAGCGGTCACGCTGCGCGT
AACCACCACACCCGCCGCGCTTAATGCGCCGCTACAGGGCGCGTCGCGCCATTCGCCATT
CAGGCTGCGCAACTGTTGGGAAGGGCGATCGGTGCGGGCCTCTTCGCTATTACGCCAGC
TGGCGAAGGGGGGATGTGCTGCAAGGCGATTAAGTTGGGTAACGCCAGGGTTTTCCCAGT
CACGACGTTGTAAAACGACGGCCAGTGAATTGTAATACGACTCACTATAGGGCGAATTGGA
GCTCCACCGCGGTGGCGGCCGCTCTAGAACTAGTGATTGTACTGAGAGTGCACCACGCTT
TTCAATTCAATTCATCATTTTTTTTTTTATTCTTTTTTTTGATTTCGGTTTCTTTGAAATTTTTTT
GATTCGGTAATCTCCGAACAGAAGGAAGAACGAAGGAAGGAGCACAGACTTAGATTGGTA
TATATACGCATATGTAGTGTTGAAGAAACATGAAATTGCCCAGTATTCTTAACCCAACTGCA
CAGAACAAAAACCTGCAGGAAACGAAGATAAATCATGTCGAAAGCTACATATAAGGAACGT
GCTGCTACTCATCCTAGTCCTGTTGCTGCCAAGCTATTTAATATCA**GTATGTTCTAGCGCTT**
GCACCATCCCATTTAACTGTAAGAAGAATTGCACGGTCCCAATTGCTCGAGACTAGTTAC
CGTTCGTATAAGAAACCATATACGAAGTTATCTCGAGAGATTTCTCTTTTACCTTTTTTTAC
TATTTTTCACTCTCCCATAACCTCCTATATTGACTGATCTGTAATAACCACGATATTATTGG
AATAAATAGGGGCTTGAAATTTGGAAAAAAAAAAAAAAACTGAAATATTTTCGTGATAAGT
GATAGTGATATTCTTCTTTTATTTGCTACTGTTACTAAGTCTCATGTACTAACATCGATTGC
TTCATTCTTTTTGTTGCTATATTATATGTTTAGTGCACGAAAAGCAAACAAACTTGTGTGCTT
CATTGGATGTTCGTACCACCAAGGAATTACTGGAGTTAGTTGAAGCATTAGGTCCCAAAATT
TGTTTACTAAAAACACATGTGGATATCTTGACTGATTTTTCCATGGAGGGCACAGTTAAGCC
GCTAAAGGCATTATCCGCCAAGTACAATTTTTTACTCTTCGAAGACAGAAAATTTGCTGACA
TTGGTAATACAGTCAAATTGCAGTACTCTGCGGGTGTATACAGAATAGCAGAATGGGCAGA
CATTACGAATGCACACGGTGTGGTGGGCCCAGGTATTGTTAGCGGTTTGAAGCAGGCGGC
AGAAGAAGTAACAAAGGAACCTAGAGGCCTTTTGATGTTAGCAGAATTGTCATGCAAGGGC
TCCCTATCTACTGGAGAATATACTAAGGGTACTGTTGACATTGCGAAGAGCGACAAAGATT
TTGTTATCGGCTTTATTGCTCAAAGAGACATGGGTGGAAGAGATGAAGGTTACGATTGGTT
GATTATGACACCCGGTGTGGGTTTAGATGACAAGGGAGACGCATTGGGTCAACAGTATAG
AACCGTGGATGATGTGGTCTCTACAGGATCTGACATTATTATTGTTGGAAGAGGACTATTTG
CAAAGGGAAGGGATGCTAAGGTAGAGGGTGAACGTTACAGAAAAGCAGGCTGGGAAGCAT
ATTTGAGAAGATGCGGCCAGCAAAACTAAAAAACTGTATTATAAGTAAATGCATGTATACTA
AACTCACAAATTAGAGCTTCAATTTAATTATATCAGTTATTACCCTATATCGACTACGTCGTA
AGGCCGTTTCTGACAGAGTAAAATTCTTGAGGGAACTTTCACCATTATGGGAAATGGTTCA
AGAAGGTATTGACTTAAACTCCATCAAATGGTCAGGTCATTGAGTGTTTTTTATTTGTTGTAT
TTTTTTTTTTTTAGAGAAAATCCTCCAATATCAAATTAGGAATCGTAGTTTCATGATTTTCTGT

TACACCTAACTTTTTGTGTGGTGCCCTCCTCCTTGTCAATATTAATGTTAAAGTGCAATTCTT
TTTCCTTATCACGTTGAGCCATTAGTATCAATTTGCTTACCTGTATTCCTTTACTATCCTCCT
TTTTCTCCTTCTTGATAAATGTATGTAGATTGCGTATATAGTTTCGTCTACCCTATGAACATA
TTCCATTTTGTAATTTCGTGTCGTTTCTATTATGAATTTCATTTATAAAGTTTATGTACAAATA
TCATAAAAAAAGAGAATCTTTTTAAGCAAGGATTTTCTTAACTTCTTCGGCGACAGCATCAC
CGACTTCGGTGGTACTGTTGGAACCACCTAAATCACCAGTTCTGATACCTGCATCCAAAAC
CTTTTTAACTGCATCTTCAATGGCCTTACCTTCTTCAGGCAAGTTCAATGACAATTTCAACAT
CATTGCAGCAGACAAGATAGTGGCGATAGGGTCAACCTTATTCTTTGGCAAATCTGGAGCA
GAACCGTGGCATGGTTCGTACAAACCAAATGCGGTGTTCTTGTCTGGCAAAGAGGCCAAG
GACGCAGATGGCAACAAACCCAAGGAACCTGGGATAACGGAGGCTTCATCGGAGATGATA
TCACCAAACATGTTGCTGGTGATTATAATACCATTTAGGTGGGTTGGGTTCTTAACTAGGAT
CATGGCGGCAGAATCAATCAATTGATGTTGAACCTTCAATGTAGGGAATTCGTTCTTGATG
GTTTCCTCCACAGTTTTTCTCCATAATCTTGAAGAGGCCAAAACATTAGCTTTATCCAAGGA
CCAAATAGGCAATGGTGGCTCATGTTGTAGGGCCATGAAAGCGGCCATTCTTGTGATTCTT
TGCACTTCTGGAACGGTGTATTGTTCACTATCCCAAGCGACACCATCACCATCGTCTTCCTT
TCTCTTACCAAAGTAAATACCTCCCACTAATTCTCTGACAACAACGAAGTCAGTACCTTTAG
CAAATTGTGGCTTGATTGGAGATAAGTCTAAAAGAGAGTCGGATGCAAAGTTACATGGTCT
TAAGTTGGCGTACAATTGAAGTTCTTTACGGATTTTTAGTAAACCTTGTTCAGGTCTAACAC
TACCGGTACCCCATTTAGGACCACCCACAGCACCTAACAAAACGGCATCAACCTTCTTGGA
GGCTTCCAGCGCCTCATCTGGAAGTGGGACACCTGTAGCATCGATAGCAGCACCACCAAT
TAAATGATTTTCGAAATCGAACTTGACATTGGAACGAACATCAGAAATAGCTTTAAGAACCT
TAATGGCTTCGGCTGTGATTTCTTGACCAACGTGGTCACCTGGCAAAACGACGATCTTCTT
AGGGGCAGACATAGGGGCAGACATTAGAATGGTATATCCTTGAAATATATATATATATTGCT
GAAATGTAAAAGGTAAGAAAAGTTAGAAAGTAAGACGATTGCTAACCACCTATTGGAAAAAA
CAATAGGTCCTTAAATAATATTGTCAACTTCAAGTATTGTGATGCAAGCATTTAGTCATGAAC
GCTTCTCTATTCTATATGAAAAGCCGGTTCCGGCCTCTCACCTTTCCTTTTTCTCCCAATTTT
TCAGTTGAAAAAGGTATATGCGTCAGGCGACCTCTGAAATTAACAAAAAATTTCCAGTCATC
GAATTTGATTCTGTGCGATAGCGCCCCTGTGTGTTCTCGTTATGTTGAGGAAAAAAATAATG
GTTGCTAAGAGATTCGAACTCTTGCATCTTACGATACCTGAGTATTCCCACAGTTAACTGCG
GTCAAGATATTTCTTGAATCAGGCGCCTTAGACCGCTCGGCCAAACAACCAATTACTTGTT
GAGAAATAGAGTATAATTATCCTATAAATATAACGTTTTTGAACACACATGAACAAGGAAGT
ACAGGACAATTGATTTTGAAGAGAATGTGGATTTTGATGTAATTGTTGGGATTCCATTTTTAA
TAAGGCAATAATATTAGGTATGTGGATATACTAGAAGTTCTCCTCGAGGGTCGATATGCGG
TGTGAGCATGCATAACTTCGTATAATGTATGCTATACGAACGGTACTCGAGGGGGGGCCC
GGTACCCAGCTTTTGTTCCCTTTAGTGAGGGTTAATTCCGAGCTTGGCGTAATCATGGTCA
TAGCTGTTTCCTGTGTGAAATTGTTATCCGCTCACAATTCCACACAACATAGGAGCCGGAA
GCATAAAGTGTAAAGCCTGGGGTGCCTAATGAGTGAGGTAACTCACATTAATTGCGTTGCG
CTCACTGCCCGCTTTCCAGTCGGGAAACCTGTCGTGCCAGCTGCATTAATGAATCGGCCA
ACGCGCGGGGAGAGGCGGTTTGCGTATTGGGCGCTCTTCCGCTTCCTCGCTCACTGACTC
GCTGCGCTCGGTCGTTCGGCTGCGGCGAGCGGTATCAGCTCACTCAAAGGCGGTAATAC
GGTTATCCACAGAATCAGGGGATAACGCAGGAAAGAACATGTGAGCAAAAGGCCAGCAAA
AGGCCAGGAACCGTAAAAAGGCCGCGTTGCTGGCGTTTTTCCATAGGCTCGGCCCCCCTG
ACGAGCATCACAAAAATCGACGCTCAAGTCAGAGGTGGCGAAACCCGACAGGACTATAAA
GATACCAGGCGTTCCCCCCTGGAAGCTCCCTCGTGCGCTCTCCTGTTCCGACCCTGCCGC

TTACCGGATACCTGTCCGCCTTTCTCCCTTCGGGAAGCGTGGCGCTTTCTCAATGCTCACG
CTGTAGGTATCTCAGTTCGGTGTAGGTCGTTCGCTCCAAGCTGGGCTGTGTGCACGAACC
CCCCGTTCAGCCCGACCGCTGCGCCTTATCCGGTAACTATCGTCTTGAGTCCAACCCGGT
AAGACACGACTTATCGCCACTGGCAGCAGCCACTGGTAACAGGATTAGCAGAGCGAGGTA
TGTAGGCGGTGCTACAGAGTTCTTGAAGTGGTGGCCTAACTACGGCTACACTAGAAGGAC
AGTATTTGGTATCTGCGCTCTGCTGAAGCCAGTTACCTTCGGAAAAAGAGTTGGTAGCTCT
TGATCCGGCAAACAAACCACCGCTGGTAGCGGTGGTTTTTTTGTTTGCAAGCAGCAGATTA
CGCGCAGAAAAAAAGGATCTCAAGAAGATCCTTTGATCTTTTCTACGGGGTCTGACGCTCA
GTGGAACGAAAACTCACGTTAAGGGATTTTGGTCATGAGATTATCAAAAAGGATCTTCACCT
AGATCCTTTTAAATTAAAAATGAAGTTTTAAATCAATCTAAAGTATATATGAGTAAACTTGGT
CTGACAGTTACCAATGCTTAATCAGTGAGGCACCTATCTCAGCGATCTGTCTATTTCGTTCA
TCCATAGTTGCCTGACTGCCCGTCGTGTAGATAACTACGATACGGGAGGGCTTACCATCTG
GCCCCAGTGCTGCAATGATACCGCGAGACCCACGCTCACCGGCTCCAGATTTATCAGCAA
TAAACCAGCCAGCCGGAAGGGCCGAGCGCAGAAGTGGTCCTGCAACTTTATCCGCCTCCA
TCCAGTCTATTAATTGTTGCCGGGAAGCTAGAGTAAGTAGTTCGCCAGTTAATAGTTTGCG
CAACGTTGTTGCCATTGCTACAGGCATCGTGGTGTCACGCTCGTCGTTTGGTATGGCTTCA
TTCAGCTCCGGTTCCCAACGATCAAGGCGAGTTACATGATCCCCCATGTTGTGAAAAAAAG
CGGTTAGCTCCTTCGGTCCTCCGATCGTTGTCAGAAGTAAGTTGGCCGCAGTGTTATCACT
CATGGTTATGGCAGCACTGCATAATTCTCTTACTGTCATGCCATCCGTAAGATGCTTTTCTG
TGACTGGTGAGTACTCAACCAAGTCATTCTGAGAATAGTGTATGCGGCGACCGAGTTGCTC
TTGCCCGGCGTCAATACGGGATAATACCGCGCCACATAGCAGAACTTTAAAAGTGCTCATC
ATTGGAAAACGTTCTTCGGGGCGAAAACTCTCAAGGATCTTACCGCTGTTGAGATCCAGTT
CGATGTAACCCACTCGTGCACCCAACTGATCTTCAGCATCTTTTACTTTCACCAGCGTTTCT
GGGTGAGCAAAAACAGGAAGGCAAAATGCCGCAAAAAAGGGAATAAGGGCGACACGGAA
ATGTTGAATACTCATACTCTTCCTTTTTCAATATTATTGAAGCATTTATCAGGGTTATTGTCT
CATGAGCGGATACATATTTGAATGTATTTAGAAAAATAAACAAATAGGGGTTCCGCGCACAT
TTCCCCGAAAAGTGCCACCTGGGTCCTTTTCATCACGTGCTATAAAAATAATTATAATTTAA
ATTTTTTAATATAAATATATAAATTAAAAATAGAAAGTAAAAAAAGAAATTAAAGAAAAAATAG
TTTTTGTTTTCCGAAGATGTAAAAGACTCTAGGGGGATCGCCAACAAATACTACCTTTTATC
TTGCTCTTCCTGCTCTCAGGTATTAATGCCGAATTGTTTCATCTTGTCTGTGTAGAAGACCA
CACACGAAAATCCTGTGATTTTACATTTTACTTATCGTTAATCGAATGTATATCTATTTAATCT
GCTTTTCTTGTCTAATAAATATATATGTAAAGTACGCTTTTTGTTGAAATTTTTTAAACCTTTG
TTTATTTTTTTTTCTTCATTCCGTAACTCTTCTACCTTCTTTATTTACTTTCTAAAATCCAAATA
CAAAACATAAAAATAAATAAACACAGAGTAAATTCCCAAATTATTCCATCATTAAAAGATACG
AGGCGCGTGTAAGTTACAGGCAAGCGATCCGTCCTAAGAAACCATTATTATCATGACATTA
ACCTATAAAAATAGGCGTATCACGAGGCCCTTTCGTC

SpeI site

5’ splice site

m2/Lox66

Branch point

The URA3 gene containing the modified actin intron (bold) is underlined.

pRC59:

TCGCGCGTTTCGGTGATGACGGTGAAAACCTCTGACACATGCAGCTCCCGGAGACGGTCA
CAGCTTGTCTGTAAGCGGATGCCGGGAGCAGACAAGCCCGTCAGGGCGCGTCAGCGGGT
GTTGGCGGGTGTCGGGGCTGGCTTAACTATGCGGCATCAGAGCAGATTGTACTGAGAGTG
CACCATAATTCCGTTTTAAGAGCTTGGTGAGCGCTAGGAGTCACTGCCAGGTATCGTTTGA
ACACGGCATTAGTCAGGGAAGTCATAACACAGTCCTTTCCCGCAATTTTCTTTTTCTATTAC
TCTTGGCCTCCTCTAGTACACTCTATATTTTTTTATGCCTCGGTAATGATTTTCATTTTTTTTT
TTCCACCTAGCGGATGACTCTTTTTTTTTCTTAGCGATTGGCATTATCACATAATGAATTATA
CATTATATAAAGTAATGTGATTTCTTCGAAGAATATACTAAAAAATGAGCAGGCAAGATAAA
CGAAGGCAAAGATGACAGAGCAGAAAGCCCTAGTAAAGCGTATTACAAATGAAACCAAGAT
TCAGATTGCGATCTCTTTAAAGGGTGGTCCCCTAGCGATAGAGCACTCGATCTTCCCAGAA
AAAGAGGCAGAAGCAGTAGCAGAACAGGCCACACAATCGCAAGTGATTAACGTCCACACA
GGTATAGGGTTTCTGGACCATATGATACATGCTCTGGCCAAGCATTCCGGCTGGTCGCTAA
TCGTTGAGTGCATTGGTGACTTACACATAGACGACCATCACACCACTGAAGACTGCGGGAT
TGCTCTCGGTCAAGCTTTTAAAGAGGCCCTACTGGCGCGTGGAGTAAAAAGGTTTGGATCA
GGATTTGCGCCTTTGGATGAGGCACTTTCCAGAGCGGTGGTAGATCTTTCGAACAGGCCG
TACGCAGTTGTCGAACTTGGTTTGCAAAGGGAGAAAGTAGGAGATCTCTCTTGCGAGATGA
TCCCGCATTTTCTTGAAAGCTTTGCAGAGGCTAGCAGAATTACCCTCCACGTTGATTGTCT
GCGAGGCAAGAATGATCATCACCGTAGTGAGAGTGCGTTCAAGGCTCTTGCGGTTGCCAT
AAGAGAAGCCACCTCGCCCAATGGTACCAACGATGTTCCCTCCACCAAAGGTGTTCTTATG
TAGTGACACCGATTATTTAAAGCTGCAGCATACGATATATATACATGTGTATATATGTATACC
TATGAATGTCAGTAAGTATGTATACGAACAGTATGATACTGAAGATGACAAGGTAATGCATC
ATTCTATACGTGTCATTCTGAACGAGGCGCGCTTTCCTTTTTTCTTTTTGCTTTTTCTTTTTTT
TTCTCTTGAACTCGACGGATCATATGCGGTGTGAAATACCGCACAGATGCGTAAGGAGAAA
ATACCGCATCAGGAAATTGTAAACGTTAATATTTTGTTAAAATTCGCGTTAAATTTTTGTTAA
ATCAGCTCATTTTTTAACCAATAGGCCGAAATCGGCAAAATCCCTTATAAATCAAAAGAATA
GACCGAGATAGGGTTGAGTGTTGTTCCAGTTTGGAACAAGAGTCCACTATTAAAGAACGTG
GACTCCAACGTCAAAGGGCGAAAAACCGTCTATCAGGGCGATGGCCCACTACGTGAACCA
TCACCCTAATCAAGTTTTTTGGGGTCGAGGTGCCGTAAAGCACTAAATCGGAACCCTAAAG
GGAGCCCCCGATTTAGAGCTTGACGGGGAAAGCCGGCGAACGTGGCGAGAAAGGAAGGG

AAGAAAGCGAAAGGAGCGGGCGCTAGGGCGCTGGCAAGTGTAGCGGTCACGCTGCGCGT
AACCACCACACCCGCCGCGCTTAATGCGCCGCTACAGGGCGCGTCGCGCCATTCGCCATT
CAGGCTGCGCAACTGTTGGGAAGGGCGATCGGTGCGGGCCTCTTCGCTATTACGCCAGC
TGGCGAAGGGGGGATGTGCTGCAAGGCGATTAAGTTGGGTAACGCCAGGGTTTTCCCAGT
CACGACGTTGTAAAACGACGGCCAGTGAATTGTAATACGACTCACTATAGGGCGAATTGGA
GCTCCACCGCGGTGGCGGCCGCTCTAGAACTAGTtaccgTTCGTATAAGAAACCATATACGA
AGTTATCTCGAGAGATTTCTCTTTTACCTTTTTTTACTATTTTTCACTCTCCCATAACCTCCTA
TATTGACTGATCTGTAATAACCACGATATTATTGGAATAAATAGGGGCTTGAAATTTGGAAA
AAAAAAAAAAACTGAAATATTTTCGTGATAAGTGATAGTGATATTCTTCTTTTATTTGCTACT
GTTACTAAGTCTCATGTACTAACATCGATTGCTTCATTCTTTTTGTTGCTATATTATATGTTTA
GTGCACGAAAAGCAAACAAACTTGTGTGCTTCATTGGATGTTCGTACCACCAAGGAATTAC
TGGAGTTAGTTGAAGCATTAGGTCCCAAAATTTGTTTACTAAAAACACATGTGGATATCTTG
ACTGATTTTTCCATGGAGGGCACAGTTAAGCCGCTAAAGGCATTATCCGCCAAGTACAATT
TTTTACTCTTCGAAGACAGAAAATTTGCTGACATTGGTAATACAGTCAAATTGCAGTACTCT
GCGGGTGTATACAGAATAGCAGAATGGGCAGACATTACGAATGCACACGGTGTGGTGGGC
CCAGGTATTGTTAGCGGTTTGAAGCAGGCGGCAGAAGAAGTAACAAAGGAACCTAGAGGC
CTTTTGATGTTAGCAGAATTGTCATGCAAGGGCTCCCTATCTACTGGAGAATATACTAAGG
GTACTGTTGACATTGCGAAGAGCGACAAAGATTTTGTTATCGGCTTTATTGCTCAAAGAGAC
ATGGGTGGAAGAGATGAAGGTTACGATTGGTTGATTATGACACCCGGTGTGGGTTTAGATG
ACAAGGGAGACGCATTGGGTCAACAGTATAGAACCGTGGATGATGTGGTCTCTACAGGAT
CTGACATTATTATTGTTGGAAGAGGACTATTTGCAAAGGGAAGGGATGCTAAGGTAGAGGG
TGAACGTTACAGAAAAGCAGGCTGGGAAGCATATTTGAGAAGATGCGGCCAGCAAAACTA
AAAAACTGTATTATAAGTAAATGCATGTATACTAAACTCACAAATTAGAGCTTCAATTTAATT
ATATCAGTTATTACCCTATATCGACTACGTCGTAAGGCCGTTTCTGACAGAGTAAAATTCTT
GAGGGAACTTTCACCATTATGGGAAATGGTTCAAGAAGGTATTGACTTAAACTCCATCAAAT
GGTCAGGTCATTGAGTGTTTTTTATTTGTTGTATTTTTTTTTTTTTTAGAGAAAATCCTCCAATA
TCAAATTAGGAATCGTAGTTTCATGATTTTCTGTTACACCTAACTTTTTGTGTGGTGCCCTCC
TCCTTGTCAATATTAATGTTAAAGTGCAATTCTTTTTCCTTATCACGTTGAGCCATTAGTATC
AATTTGCTTACCTGTATTCCTTTACTATCCTCCTTTTTCTCCTTCTTGATAAATGTATGTAGAT
TGCGTATATAGTTTCGTCTACCCTATGAACATATTCCATTTTGTAATTTCGTGTCGTTTCTAT
TATGAATTTCATTTATAAAGTTTATGTACAAATATCATAAAAAAAGAGAATCTTTTTAAGCAAG
GATTTTCTTAACTTCTTCGGCGACAGCATCACCGACTTCGGTGGTACTGTTGGAACCACCT
AAATCACCAGTTCTGATACCTGCATCCAAAACCTTTTTAACTGCATCTTCAATGGCCTTACC
TTCTTCAGGCAAGTTCAATGACAATTTCAACATCATTGCAGCAGACAAGATAGTGGCGATA
GGGTCAACCTTATTCTTTGGCAAATCTGGAGCAGAACCGTGGCATGGTTCGTACAAACCAA
ATGCGGTGTTCTTGTCTGGCAAAGAGGCCAAGGACGCAGATGGCAACAAACCCAAGGAAC
CTGGGATAACGGAGGCTTCATCGGAGATGATATCACCAAACATGTTGCTGGTGATTATAAT
ACCATTTAGGTGGGTTGGGTTCTTAACTAGGATCATGGCGGCAGAATCAATCAATTGATGT
TGAACCTTCAATGTAGGGAATTCGTTCTTGATGGTTTCCTCCACAGTTTTTCTCCATAATCTT
GAAGAGGCCAAAACATTAGCTTTATCCAAGGACCAAATAGGCAATGGTGGCTCATGTTGTA
GGGCCATGAAAGCGGCCATTCTTGTGATTCTTTGCACTTCTGGAACGGTGTATTGTTCACT
ATCCCAAGCGACACCATCACCATCGTCTTCCTTTCTCTTACCAAAGTAAATACCTCCCACTA
ATTCTCTGACAACAACGAAGTCAGTACCTTTAGCAAATTGTGGCTTGATTGGAGATAAGTCT
AAAAGAGAGTCGGATGCAAAGTTACATGGTCTTAAGTTGGCGTACAATTGAAGTTCTTTAC

GGATTTTTAGTAAACCTTGTTCAGGTCTAACACTACCGGTACCCCATTTAGGACCACCCACA
GCACCTAACAAAACGGCATCAACCTTCTTGGAGGCTTCCAGCGCCTCATCTGGAAGTGGG
ACACCTGTAGCATCGATAGCAGCACCACCAATTAAATGATTTTCGAAATCGAACTTGACATT
GGAACGAACATCAGAAATAGCTTTAAGAACCTTAATGGCTTCGGCTGTGATTTCTTGACCAA
CGTGGTCACCTGGCAAAACGACGATCTTCTTAGGGGCAGACATAGGGGCAGACATTAGAA
TGGTATATCCTTGAAATATATATATATATTGCTGAAATGTAAAAGGTAAGAAAAGTTAGAAAG
TAAGACGATTGCTAACCACCTATTGGAAAAAACAATAGGTCCTTAAATAATATTGTCAACTT
CAAGTATTGTGATGCAAGCATTTAGTCATGAACGCTTCTCTATTCTATATGAAAAGCCGGTT
CCGGCCTCTCACCTTTCCTTTTTCTCCCAATTTTTCAGTTGAAAAAGGTATATGCGTCAGGC
GACCTCTGAAATTAACAAAAAATTTCCAGTCATCGAATTTGATTCTGTGCGATAGCGCCCCT
GTGTGTTCTCGTTATGTTGAGGAAAAAAATAATGGTTGCTAAGAGATTCGAACTCTTGCATC
TTACGATACCTGAGTATTCCCACAGTTAACTGCGGTCAAGATATTTCTTGAATCAGGCGCCT
TAGACCGCTCGGCCAAACAACCAATTACTTGTTGAGAAATAGAGTATAATTATCCTATAAAT
ATAACGTTTTTGAACACACATGAACAAGGAAGTACAGGACAATTGATTTTGAAGAGAATGTG
GATTTTGATGTAATTGTTGGGATTCCATTTTTAATAAGGCAATAATATTAGGTATGTGGATAT
ACTAGAAGTTCTCCTCGAGGGTCGATATGCGGTGTGAGCATGCATAACTTCGTATAATGTA
TGCTATACGAAcggtaCTCGAGGGGGGGCCCGGTACCCAGCTTTTGTTCCCTTTAGTGAGGG
TTAATTCCGAGCTTGGCGTAATCATGGTCATAGCTGTTTCCTGTGTGAAATTGTTATCCGCT
CACAATTCCACACAACATAGGAGCCGGAAGCATAAAGTGTAAAGCCTGGGGTGCCTAATG
AGTGAGGTAACTCACATTAATTGCGTTGCGCTCACTGCCCGCTTTCCAGTCGGGAAACCTG
TCGTGCCAGCTGCATTAATGAATCGGCCAACGCGCGGGGAGAGGCGGTTTGCGTATTGG
GCGCTCTTCCGCTTCCTCGCTCACTGACTCGCTGCGCTCGGTCGTTCGGCTGCGGCGAGC
GGTATCAGCTCACTCAAAGGCGGTAATACGGTTATCCACAGAATCAGGGGATAACGCAGG
AAAGAACATGTGAGCAAAAGGCCAGCAAAAGGCCAGGAACCGTAAAAAGGCCGCGTTGCT
GGCGTTTTTCCATAGGCTCGGCCCCCCTGACGAGCATCACAAAAATCGACGCTCAAGTCA
GAGGTGGCGAAACCCGACAGGACTATAAAGATACCAGGCGTTCCCCCCTGGAAGCTCCCT
CGTGCGCTCTCCTGTTCCGACCCTGCCGCTTACCGGATACCTGTCCGCCTTTCTCCCTTCG
GGAAGCGTGGCGCTTTCTCAATGCTCACGCTGTAGGTATCTCAGTTCGGTGTAGGTCGTTC
GCTCCAAGCTGGGCTGTGTGCACGAACCCCCCGTTCAGCCCGACCGCTGCGCCTTATCC
GGTAACTATCGTCTTGAGTCCAACCCGGTAAGACACGACTTATCGCCACTGGCAGCAGCC
ACTGGTAACAGGATTAGCAGAGCGAGGTATGTAGGCGGTGCTACAGAGTTCTTGAAGTGG
TGGCCTAACTACGGCTACACTAGAAGGACAGTATTTGGTATCTGCGCTCTGCTGAAGCCAG
TTACCTTCGGAAAAAGAGTTGGTAGCTCTTGATCCGGCAAACAAACCACCGCTGGTAGCG
GTGGTTTTTTTGTTTGCAAGCAGCAGATTACGCGCAGAAAAAAAGGATCTCAAGAAGATCC
TTTGATCTTTTCTACGGGGTCTGACGCTCAGTGGAACGAAAACTCACGTTAAGGGATTTTG
GTCATGAGATTATCAAAAAGGATCTTCACCTAGATCCTTTTAAATTAAAAATGAAGTTTTAAA
TCAATCTAAAGTATATATGAGTAAACTTGGTCTGACAGTTACCAATGCTTAATCAGTGAGGC
ACCTATCTCAGCGATCTGTCTATTTCGTTCATCCATAGTTGCCTGACTGCCCGTCGTGTAGA
TAACTACGATACGGGAGGGCTTACCATCTGGCCCCAGTGCTGCAATGATACCGCGAGACC
CACGCTCACCGGCTCCAGATTTATCAGCAATAAACCAGCCAGCCGGAAGGGCCGAGCGCA
GAAGTGGTCCTGCAACTTTATCCGCCTCCATCCAGTCTATTAATTGTTGCCGGGAAGCTAG
AGTAAGTAGTTCGCCAGTTAATAGTTTGCGCAACGTTGTTGCCATTGCTACAGGCATCGTG
GTGTCACGCTCGTCGTTTGGTATGGCTTCATTCAGCTCCGGTTCCCAACGATCAAGGCGA
GTTACATGATCCCCCATGTTGTGAAAAAAAGCGGTTAGCTCCTTCGGTCCTCCGATCGTTG

TCAGAAGTAAGTTGGCCGCAGTGTTATCACTCATGGTTATGGCAGCACTGCATAATTCTCTT
ACTGTCATGCCATCCGTAAGATGCTTTTCTGTGACTGGTGAGTACTCAACCAAGTCATTCTG
AGAATAGTGTATGCGGCGACCGAGTTGCTCTTGCCCGGCGTCAATACGGGATAATACCGC
GCCACATAGCAGAACTTTAAAAGTGCTCATCATTGGAAAACGTTCTTCGGGGCGAAAACTC
TCAAGGATCTTACCGCTGTTGAGATCCAGTTCGATGTAACCCACTCGTGCACCCAACTGAT
CTTCAGCATCTTTTACTTTCACCAGCGTTTCTGGGTGAGCAAAAACAGGAAGGCAAAATGC
CGCAAAAAAGGGAATAAGGGCGACACGGAAATGTTGAATACTCATACTCTTCCTTTTTCAAT
ATTATTGAAGCATTTATCAGGGTTATTGTCTCATGAGCGGATACATATTTGAATGTATTTAGA
AAAATAAACAAATAGGGGTTCCGCGCACATTTCCCCGAAAAGTGCCACCTGGGTCCTTTTC
ATCACGTGCTATAAAAATAATTATAATTTAAATTTTTTAATATAAATATATAAATTAAAAATAGA
AAGTAAAAAAAGAAATTAAAGAAAAAATAGTTTTTGTTTTCCGAAGATGTAAAAGACTCTAGG
GGGATCGCCAACAAATACTACCTTTTATCTTGCTCTTCCTGCTCTCAGGTATTAATGCCGAA
TTGTTTCATCTTGTCTGTGTAGAAGACCACACACGAAAATCCTGTGATTTTACATTTTACTTA
TCGTTAATCGAATGTATATCTATTTAATCTGCTTTTCTTGTCTAATAAATATATATGTAAAGTA
CGCTTTTTGTTGAAATTTTTTAAACCTTTGTTTATTTTTTTTTCTTCATTCCGTAACTCTTCTAC
CTTCTTTATTTACTTTCTAAAATCCAAATACAAAACATAAAAATAAATAAACACAGAGTAAATT
CCCAAATTATTCCATCATTAAAAGATACGAGGCGCGTGTAAGTTACAGGCAAGCGATCCGT
CCTAAGAAACCATTATTATCATGACATTAACCTATAAAAATAGGCGTATCACGAGGCCCTTT
CGTC

pRC60:

ATCGATACCGTCGACCTCGAGGGGGGGCCCGGTACCCAGCTTTTGTTCCCTTTAGTGAGG
GTTAATTCCGAGCTTGGCGTAATCATGGTCATAGCTGTTTCCTGTGTGAAATTGTTATCCGC
TCACAATTCCACACAACATAGGAGCCGGAAGCATAAAGTGTAAAGCCTGGGGTGCCTAATG
AGTGAGGTAACTCACATTAATTGCGTTGCGCTCACTGCCCGCTTTCCAGTCGGGAAACCTG
TCGTGCCAGCTGCATTAATGAATCGGCCAACGCGCGGGGAGAGGCGGTTTGCGTATTGG
GCGCTCTTCCGCTTCCTCGCTCACTGACTCGCTGCGCTCGGTCGTTCGGCTGCGGCGAGC
GGTATCAGCTCACTCAAAGGCGGTAATACGGTTATCCACAGAATCAGGGGATAACGCAGG
AAAGAACATGTGAGCAAAAGGCCAGCAAAAGGCCAGGAACCGTAAAAAGGCCGCGTTGCT
GGCGTTTTTCCATAGGCTCGGCCCCCCTGACGAGCATCACAAAAATCGACGCTCAAGTCA
GAGGTGGCGAAACCCGACAGGACTATAAAGATACCAGGCGTTCCCCCCTGGAAGCTCCCT
CGTGCGCTCTCCTGTTCCGACCCTGCCGCTTACCGGATACCTGTCCGCCTTTCTCCCTTCG
GGAAGCGTGGCGCTTTCTCAATGCTCACGCTGTAGGTATCTCAGTTCGGTGTAGGTCGTTC
GCTCCAAGCTGGGCTGTGTGCACGAACCCCCCGTTCAGCCCGACCGCTGCGCCTTATCC
GGTAACTATCGTCTTGAGTCCAACCCGGTAAGACACGACTTATCGCCACTGGCAGCAGCC
ACTGGTAACAGGATTAGCAGAGCGAGGTATGTAGGCGGTGCTACAGAGTTCTTGAAGTGG
TGGCCTAACTACGGCTACACTAGAAGGACAGTATTTGGTATCTGCGCTCTGCTGAAGCCAG
TTACCTTCGGAAAAAGAGTTGGTAGCTCTTGATCCGGCAAACAAACCACCGCTGGTAGCG
GTGGTTTTTTTGTTTGCAAGCAGCAGATTACGCGCAGAAAAAAAGGATCTCAAGAAGATCC
TTTGATCTTTTCTACGGGGTCTGACGCTCAGTGGAACGAAAACTCACGTTAAGGGATTTTG
GTCATGAGATTATCAAAAAGGATCTTCACCTAGATCCTTTTAAATTAAAAATGAAGTTTTAAA
TCAATCTAAAGTATATATGAGTAAACTTGGTCTGACAGTTACCAATGCTTAATCAGTGAGGC
ACCTATCTCAGCGATCTGTCTATTTCGTTCATCCATAGTTGCCTGACTGCCCGTCGTGTAGA
TAACTACGATACGGGAGGGCTTACCATCTGGCCCCAGTGCTGCAATGATACCGCGAGACC

CACGCTCACCGGCTCCAGATTTATCAGCAATAAACCAGCCAGCCGGAAGGGCCGAGCGCA
GAAGTGGTCCTGCAACTTTATCCGCCTCCATCCAGTCTATTAATTGTTGCCGGGAAGCTAG
AGTAAGTAGTTCGCCAGTTAATAGTTTGCGCAACGTTGTTGCCATTGCTACAGGCATCGTG
GTGTCACGCTCGTCGTTTGGTATGGCTTCATTCAGCTCCGGTTCCCAACGATCAAGGCGA
GTTACATGATCCCCCATGTTGTGAAAAAAAGCGGTTAGCTCCTTCGGTCCTCCGATCGTTG
TCAGAAGTAAGTTGGCCGCAGTGTTATCACTCATGGTTATGGCAGCACTGCATAATTCTCTT
ACTGTCATGCCATCCGTAAGATGCTTTTCTGTGACTGGTGAGTACTCAACCAAGTCATTCTG
AGAATAGTGTATGCGGCGACCGAGTTGCTCTTGCCCGGCGTCAATACGGGATAATACCGC
GCCACATAGCAGAACTTTAAAAGTGCTCATCATTGGAAAACGTTCTTCGGGGCGAAAACTC
TCAAGGATCTTACCGCTGTTGAGATCCAGTTCGATGTAACCCACTCGTGCACCCAACTGAT
CTTCAGCATCTTTTACTTTCACCAGCGTTTCTGGGTGAGCAAAAACAGGAAGGCAAAATGC
CGCAAAAAAGGGAATAAGGGCGACACGGAAATGTTGAATACTCATACTCTTCCTTTTTCAAT
ATTATTGAAGCATTTATCAGGGTTATTGTCTCATGAGCGGATACATATTTGAATGTATTTAGA
AAAATAAACAAATAGGGGTTCCGCGCACATTTCCCCGAAAAGTGCCACCTGGGTCCTTTTC
ATCACGTGCTATAAAAATAATTATAATTTAAATTTTTTAATATAAATATATAAATTAAAAATAGA
AAGTAAAAAAAGAAATTAAAGAAAAAATAGTTTTTGTTTTCCGAAGATGTAAAAGACTCTAGG
GGGATCGCCAACAAATACTACCTTTTATCTTGCTCTTCCTGCTCTCAGGTATTAATGCCGAA
TTGTTTCATCTTGTCTGTGTAGAAGACCACACACGAAAATCCTGTGATTTTACATTTTACTTA
TCGTTAATCGAATGTATATCTATTTAATCTGCTTTTCTTGTCTAATAAATATATATGTAAAGTA
CGCTTTTTGTTGAAATTTTTTAAACCTTTGTTTATTTTTTTTTCTTCATTCCGTAACTCTTCTAC
CTTCTTTATTTACTTTCTAAAATCCAAATACAAAACATAAAAATAAATAAACACAGAGTAAATT
CCCAAATTATTCCATCATTAAAAGATACGAGGCGCGTGTAAGTTACAGGCAAGCGATCCGT
CCTAAGAAACCATTATTATCATGACATTAACCTATAAAAATAGGCGTATCACGAGGCCCTTT
CGTCTCGCGCGTTTCGGTGATGACGGTGAAAACCTCTGACACATGCAGCTCCCGGAGACG
GTCACAGCTTGTCTGTAAGCGGATGCCGGGAGCAGACAAGCCCGTCAGGGCGCGTCAGC
GGGTGTTGGCGGGTGTCGGGGCTGGCTTAACTATGCGGCATCAGAGCAGATTGTACTGAG
AGTGCACCATAAACGACATTACTATATATATAATATAGGAAGCATTTAATAGACAGCATCGT
AATATATGTGTACTTTGCAGTTATGACGCCAGATGGCAGTAGTGGAAGATATTCTTTATTGA
AAAATAGCTTGTCACCTTACGTACAATCTTGATCCGGAGCTTTTCTTTTTTTGCCGATTAAGA
ATTAATTCGGTCGAAAAAAGAAAAGGAGAGGGCCAAGAGGGAGGGCATTGGTGACTATTG
AGCACGTGAGTATACGTGATTAAGCACACAAAGGCAGCTTGGAGTATGTCTGTTATTAATTT
CACAGGTAGTTCTGGTCCATTGGTGAAAGTTTGCGGCTTGCAGAGCACAGAGGCCGCAGA
ATGTGCTCTAGATTCCGATGCTGACTTGCTGGGTATTATATGTGTGCCCAATAGAAAGAGA
ACAATTGACCCGGTTATTGCAAGGAAAATTTCAAGTCTTGTAAAAGCATATAAAAATAGTTC
AGGCACTCCGAAATACTTGGTTGGCGTGTTTCGTAATCAACCTAAGGAGGATGTTTTGGCT
CTGGTCAATGATTACGGCATTGATATCGTCCAACTGCATGGAGATGAGTCGTGGCAAGAAT
ACCAAGAGTTCCTCGGTTTGCCAGTTATTAAAAGACTCGTATTTCCAAAAGACTGCAACATA
CTACTCAGTGCAGCTTCACAGAAACCTCATTCGTTTATTCCCTTGTTTGATTCAGAAGCAGG
TGGGACAGGTGAACTTTTGGATTGGAACTCGATTTCTGACTGGGTTGGAAGGCAAGAGAG
CCCCGAAAGCTTACATTTTATGTTAGCTGGTGGACTGACGCCAGAAAATGTTGGTGATGCG
CTTAGATTAAATGGCGTTATTGGTGTTGATGTAAGCGGAGGTGTGGAGACAAATGGTGTAA
AAGACTCTAACAAAATAGCAAATTTCGTCAAAAATGCTAAGAAATAGGTTATTACTGAGTAG
TATTTATTTAAGTATTGTTTGTGCACTTGCCTGCGGTGTGAAATACCGCACAGATGCGTAAG
GAGAAAATACCGCATCAGGAAATTGTAAACGTTAATATTTTGTTAAAATTCGCGTTAAATTTT

TGTTAAATCAGCTCATTTTTTAACCAATAGGCCGAAATCGGCAAAATCCCTTATAAATCAAAA
GAATAGACCGAGATAGGGTTGAGTGTTGTTCCAGTTTGGAACAAGAGTCCACTATTAAAGA
ACGTGGACTCCAACGTCAAAGGGCGAAAAACCGTCTATCAGGGCGATGGCCCACTACGTG
AACCATCACCCTAATCAAGTTTTTTGGGGTCGAGGTGCCGTAAAGCACTAAATCGGAACCC
TAAAGGGAGCCCCCGATTTAGAGCTTGACGGGGAAAGCCGGCGAACGTGGCGAGAAAGG
AAGGGAAGAAAGCGAAAGGAGCGGGCGCTAGGGCGCTGGCAAGTGTAGCGGTCACGCTG
CGCGTAACCACCACACCCGCCGCGCTTAATGCGCCGCTACAGGGCGCGTCGCGCCATTC
GCCATTCAGGCTGCGCAACTGTTGGGAAGGGCGATCGGTGCGGGCCTCTTCGCTATTACG
CCAGCTGGCGAAGGGGGGATGTGCTGCAAGGCGATTAAGTTGGGTAACGCCAGGGTTTT
CCCAGTCACGACGTTGTAAAACGACGGCCAGTGAATTGTAATACGACTCACTATAGGGCGA
ATTGGAGCTCCACCGCGGTGGCGGCCGCTCTAGAACTAGTGATTGTACTGAGAGTGCACC
ACGCTTTTCAATTCAATTCATCATTTTTTTTTTATTCTTTTTTTTGATTTCGGTTTCTTTGAAAT
TTTTTTGATTCGGTAATCTCCGAACAGAAGGAAGAACGAAGGAAGGAGCACAGACTTAGAT
TGGTATATATACGCATATGTAGTGTTGAAGAAACATGAAATTGCCCAGTATTCTTAACCCAA
CTGCACAGAACAAAAACCTGCAGGAAACGAAGATAAATCATGTCGAAAGCTACATATAAGG
AACGTGCTGCTACTCATCCTAGTCCTGTTGCTGCCAAGCTATTTAATATCAGTATGTTCTAG
CGCTTGCACCATCCCATTTAACTGTAAGAAGAATTGCACGGTCCCAATTGCTCGAGACTAG
TATAACTTCGTATAAGAAACCATATACGAAcggtaCTGCAGATGGCTACTAATATTACTTGGCA
TCCAAATCTTACTTACGACGAACGCAAGGCATTGAGAAAACAGGACGGTTGTACTATTTGG
TTAACAGGTCTAAGTGCGTCAGGTAAAAGTACAATCGCCTGTGCGCTAGAACAGTTACTGC
TCCAAAAAAACTTGTCTGCATATAGATTGGATGGTGACAACATTCGTTTTGGATTGAACAAG
GATTTGGGTTTCTCAGAAAAGGACAGAAATGAAAACATTCGTAGAATTAGCGAAGTTTCTAA
GCTATTTGCTGATTCATGTGCTATTTCAATCACCTCATTTATCTCTCCATACAGAGTTGACAG
AGATAGAGCTCGTGAACTACATAAGGAGGCTGGTTTGAAGTTCATTGAAATATTTGTTGATG
TTCCATTAGAAGTCGCTGAGCAAAGGGACCCTAAGGGTTTATACAAGAAAGCTAGGGAGG
GTGTAATCAAGGAGTTTACAGGTATTTCTGCCCCATATGAAGCGCCAAAAGCTCCAGAGCT
ACATTTGAGAACCGACCAGAAGACGGTTGAAGAATGTGCTACCATTATTTATGAGTACTTAA
TCAGTGAAAAAATCATCCGTAAGCATTTGTAACTGCAGtaccgTTCGTATAATGTATGCTATAC
GAAGTTATGCATGCCTGTTCGGAGATTACCGAATCAAAAAAATTTCAAAGAAACCGAAATCA
ACTAATCGCCATCTTCCAGCAGGCGCACCATTGCCCCTGTTTCACTATCCAGGTTACGGAT
ATAGTTCATGACAATATTTACATTGGTCCAGCCACCAGCTTGCATGATCTCCGGTATTGAAA
CTCCAGCGCGGGCCATATCTCGCGCGGCTCCGACACGGGCACTGTGTCCAGACCAGGCC
AGGTATCTCTGACCAGAGTCATCCTTAGCGCCGTAAATCAATCGATGAGTTGCTTCAAAAAT
CCCTTCCAGGGCGCGAGTTGATAGCTGGCTGGTGGCAGATGGCGCGGCAACACCATTTTT
TCTGACCCGGCAAAACAGGTAGTTATTCGGATCATCAGCTACACCAGAGACGGAAATCCAT
CGCTCGACCAGTTTAGTTACCCCCAGGCTAAGTGCCTTCTCTACACCTGCGGTGCTAACCA
GCGTTTTCGTTCTGCCAATATGGATTAACATTCTCCCACCGTCAGTACGTGAGATATCTTTA
ACCCTGATCCTGGCAATTTCGGCTATACGTAACAGGGTGTTATAAGCAATCCCCAGAAATG
CCAGATTACGTATATCCTGGCAGCGATCGCTATTTTCCATGAGTGAACGAACCTGGTCGAA
ATCAGTGCGTTCGAACGCTAGAGCCTGTTTTGCACGTTCACCGGCATCAACGTTTTCTTTT
CGGATCCGCCGCATAACCAGTGAAACAGCATTGCTGTCACTTGGTCGTGGCAGCCCGGAC
CGACGATGAAGCATGTTTAGCTGGCCCAAATGTTGCTGGATAGTTTTTACTGCCAGACCGC
GCGCCTGAAGATATAGAAGATAATCGCGAACATCTTCAGGTTCTGCGGGAAACCATTTCCG
GTTATTCAACTTGCACCATGCCGCCCACGACCGGCAAACGGACAGAAGCATTTTCCAGGTA

TGCTCAGAAAACGCCTGGCGATCCCTGAACATGTCCATCAGGTTCTTGCGAACCTCATCAC
TCGTTGCATCGACCGGTAATGCAGGCAAATTTTGGTGTACGGTCAGTAAATTGGACATGGT
TTTTTCTCCTTGACGTTAAAGTATAGAGGTATATTAACAATTTTTTGTTGATACTTTTATTACA
TTTGAATAAGAAGTAATACAAACCGAAAATGTTGAAAGTATTAGTTAAAGTGGTTAATGCAG
TTTTTGCATTTATATATCTGTTAATAGATCAAAAATCATCGCTTCGCTGATTAATTACCCCAG
AAATAAGGCTAAAAAACTAATCGCATTATCATCCTATGGTTGTTAATTTGATTCGTTCATTTG
AAGGTTTGTGGGGCCAGGTTACTGCCAATTTTTCCTCTTCATAACCATAAAAGCTAGTATTG
TAGAATCTTTATTGTTCGGAGCAGTGCGGCGCGAGGCACATCTGCGTTTCAGGAACGCGA
CCGGTGAGGACGAGGACGCACGGAGGAGAGTCTTCCTTCGGAGGGCTGTCACCCGCTCG
GCGGCTTCTAATCCGT
